# Detection of IL-1β, VEGF and IL-4 with their novel genetic variations in breast cancer patients

**DOI:** 10.1016/j.sjbs.2022.103544

**Published:** 2022-12-23

**Authors:** Tara Farooq Mohammed, Fikry Ali Qadir

**Affiliations:** Biology Department, College of Science, Salahaddin University, Erbil, Iraq

**Keywords:** Interleukin-1β, Interleukin-4, VEGF, Breast Cancer

## Abstract

Interleukin-1β (IL-1β), vascular endothelial growth factor (VEGF), and IL-4 serum levels and new genetic mutations in breast cancer (BC) patients were assessed in the current study. The serum levels of the examined cytokines in 40 BC patients and 40 control subjects were assessed using the ELISA technique. In order to identify genotype variants of the IL-1β, IL-4, and VEGF genes in 40 Formalin Fixed Paraffin Embedded (FFPE) samples with BC and 10 FFPE samples from healthy women's breast tissue, Sanger sequencing was used. According to this study, BC patients had significantly lower serum concentrations of IL-4 and significantly higher quantities of the tumor markers, CA15-3, IL-1β, and VEGF. In terms of genotype alterations, a total of 21 mutations in three trialed genes (eight in IL-1β, 10 in IL-4, and three in VEGF) were found in BC patients. The results of the current investigation suggested that angiogenesis and the development of BC may be significantly influenced by the genetic differences and higher levels of the examined cytokines.

## 1 Introduction

Globally, breast cancer is the most rampant cancer as compared with other malignancies such as lung cancer, colorectal cancer, prostate cancer, and stomach cancer. It is the most recurrently detected cancer among women in the world (Siegel et al., 2018, Chen et al., 2016). In this regard, breast cancer's initiation, development, progression and metastasis are intricate biologically, immunologically, hormonally, and molecularly. Having said, many intrinsic and extrinsic factors have pivotal roles in developing breast cancer in females (Anderson et al., 2014).

According to data published in 2022 breast and lung cancer were the most frequent cancers in the Erbil and Duhok governorates in northern Iraq between 2013 and 2019 (Karwan et al., 2022). It is believed that specific genetic variations have an impact on how proteins function, mRNA integrity, and gene transcription (Rannala, 2001). In an effort to better reliably stratify BC risk; a lot of researches have been done on the genetic variation linked to the disease (Wang et al., 2017, Adab et al., 2017). Multifunctional cytokines involved in inflammatory and immunological responses are strongly linked to the etiology of malignant disorders, making them potential concerns for BC (Mendez-García et al., 2019). The immune system and cancer cells interact heavily throughout the development of tumors. In fact, different cancers contain different types of immune cells (Barnes and Amir, 2017).

Tumor markers are frequently used in cancer surveillance and screening. Cancer Antigen 15-3 (CA153), a tumor marker, has received FDA approval for use in the monitoring of BC (Uehara et al., 2008, O'Hanlon et al., 1995). The mucinous glycoprotein CA15-3 is a product of the Mucin1 (MUC-1) gene and is one of its byproducts (Thompson et al., 1991). MUC-1 is present in almost all epithelial cells, and its overexpression has frequently been associated with colon, breast, ovarian, lung, and pancreatic malignancies (Gendler, 2001, Roy et al., 2011). Previous studies have shown that high serum CA15-3 levels are commonly detected in BC patients (Park et al., 2008, Metwally et al., 2011, Agha-Hosseini et al., 2009).

On chromosome 2q14.2, the interleukin 1 gene family contains the genes for IL1-α, IL-1β, and IL-1 receptor antagonist (IL-RA) (Bensen et al., 2001). The proinflammatory cytokines IL1-α and IL-1β are potent, but IL-RA is an anti-inflammatory cytokine (Dinarello, 1996). IL-1β is a cytokine that can be produced by different cells that modifies the host's reaction to microbial invasion, tissue injury, and inflammation (Dinarello, 2013).

Th2 production of the crucial differentiation cytokine interleukin 4 (IL-4) drives the development of Th2. By activating granulocytes and eosinophils and inhibiting angiogenesis, the Th2 subset of lymphocytes is responsible for the elimination of tumor cells (Salimi et al., 2014). In addition, some studies have shown that the Th2 subset inhibits macrophage activation, antagonizes IFN-γ, and has anti-tumor effects on a number of malignancies including renal, BC, and colon (Alshaker and Matalka, 2011). The IL-4 gene is located on chromosome 5 and has a 70-bp VNTR polymorphism inside intron 3 with two common alleles (Kok et al., 2017).

An important stage in the development of a malignant tumor is angiogenesis. VEGF is an important factor in angiogenesis because it is a potent endothelial cell activator. An advanced stage of the disease and a bad prognosis are associated with elevated levels of VEGF and an increased density of microvessels in several types of malignant tumors, including BC (Abou Shousha et al., 2022). The VEGF gene has 8 exons and is found on chromosome 6p21.3 (Vincenti et al., 1996). Variants in the VEGF gene have been identified and connected to a higher risk of a number of malignancies (Chand Bayal et al., 2021, Guleria et al., 2022).

Single nucleotide polymorphisms (SNPs), which have been shown to alter the expression or function of cytokines, play a key role in the development and progression of BC (Brentnall et al., 2020). Cytokines have been associated with cancer immunotherapy and carcinogenesis (Hirotsu et al., 2015). SNPs in the related protein's intron, exon, promoter, or untranslated regions may have an impact on production or even function. Numerous SNPs in the VEGF gene were discovered to be associated with variable VEGF expression (Albalawi et al., 2020, Li et al., 2021).

Since there are not enough studies on the immunological variations concerning patients with BC in northern Iraq, this study set out to determine the alterations in IL-1-β, IL-4, and VEGF serum concentrations as well as the genetic polymorphisms of the IL-1-β, IL-4, and VEGF genes in BC patients.

## 2 Materials and methods

### 2.1 Patient and sample collection

The Rizgary, PAR, and Nanakaly Hospitals' oncology departments as well as the Al-Mufti private laboratory in the city of Erbil are where the study's sample collection took place. This study was carried out in accordance with the principles of the Helsinki Declaration and was approved by the Human Ethics Committee of the Science College at Salahaddin University Erbil (No:45/501; date, October 24, 2021; Erbil, Iraq). Any participant with previous cancer, even if anyone in their family had cancer, or there was any hereditary disease, smokers, other types of cancer, autoimmune disease, or who’s under chemotherapy were excluded from the study. The venous blood samples are taken from 40 female patients with BC, with mean ages (53.29 ± 1.76) years; and 40 female controls, with mean ages (48 ± 1.83) years. In order to create FFPE specimen blocks, specimen blocks from female BC patients and female controls were used. The collection of FFPE and blood samples took place between November 2021 and April 2022.

### 2.2 Blood sample collection

Both control cases and BC patients' blood was drawn into a 5-ml syringe, and then 3 ml for cytokines and 2 ml for the tumor marker CA15-3 were added to each of two tubes of yellow, non-heparinized gel. After being centrifuged at 5000 rpm for 10 min to prepare the serum for cytokine evaluation, blood samples were immediately sent to the laboratory. The serum was then transferred to a 1.5 ml Eppendorf tube and stored in a deep freezer at (−80 °C) for use in cytokine concentration measurement.

### 2.3 Determination of tumor marker Ca15-3

Two ml blood were collected by syringe from both patients BC and control cases in Oncology department of Nanakaly and Rizgary Hospital in Erbil city, and added to the non-Heparinized gel tube after they clotted directly transferred to the Smart private Laboratory in Erbil city and then serum was prepared by 5000 rpm centrifuge for 10 min. Finally, evaluation of CA15-3 was done by diagnostic reagent Kit (Roch CA15-3, Germany) and Cobas e411 used for reading (Roch, Germany).

### 2.4 Determinations of IL-4, IL-1β and VGEF concertation

Human IL-1β ELISA Kit (cat. no. SL0984Hu), IL-4 ELISA Kit (cat. no. SL0997Hu), and VEGF ELISA Kit (cat. no. SL1811Hu) from are used to measure the serum concentrations of cytokines (Sunlong Biotech Co., Ltd). Sandwich-ELISA was the assessment methodology employed in these ELISA kits. By using an ELISA reader, the optical density (OD) was determined spectrophotometrically at wavelengths of 450 nm (BioTek Instruments, Inc.). The OD values correlate with the levels of IL-1β, IL-4, and VEGF. The OD of the samples was compared to the reference curves to determine the amounts of IL-1β, IL-4, and VEGF.

### 2.5 DNA extraction

The genomic DNA was extracted using the GeneRead DNA FFPE Kit (Cat. No. 180134, Germany) from GIAGEN from FFPE blocks that were verified histopathologically to contain malignant tumor of BC. The A260/A280 ratio and eluted DNA quantities in ng/L were assessed using a Nanodrop spectrophotometer (Biometrics; OneDrop TOUCH). The Nanodrop instrument was blanked using the elution buffer ATE. The optimal A260/A280 ratio was used to calculate the DNA's quantity and purity.

### 2.6 Genotype determinations

The three genes examined in the current study are IL-1β, IL-4, and VEGF. Three alternative locations were chosen based on previously published datasets (Eras et al., 2019, Chu et al., 2020, Rezaei et al., 2016). These Promoter region of included IL-1 (product length 240 nt), IL-4 (product length 334 nt), and 3′-UTR region of VEGF (product length 443nt). Each gene's pure DNA was first amplified using a ready-to-use master mix from ADDBIO Inc. Thermocycler PCR was employed with an Eppendorf, vapo. Protect, and BIO RAD C1000 equipment for each genetic polymorphism. The following primers were used; IL-1β (rs1143627) Forward, 5′-AGAAGCTTCCACCAATACT 3′, and Reverse, 5′ TAGCACCTAGTTGTAAGGA 3׳; IL-4 (rs2070874) Forward, 5׳ CTGGAAGAGAGGTGCTGATT 3׳, and Reverse, 5׳ ACTCACCTTCTGCTCTGTGA 3׳; and VEGF (rs3025039) Forward, 5׳ACACCATCACCATCGACAGA 3′and Reverse 5′TCTCCTCCTCTTCCCTGTCA 3′ (Integrated DNA technologies, Canada). The following PCR-thermocycling conditions were used initial denaturation 5 min at 95 °C; followed via 35 cycles at 95 °C of 25 s for (IL-1β, IL-4), and (VEGF) followed via 30 cycles at 95 °C of 30 s, annealing stepped 45.5 °C of 30 s for (IL1-β), At 52.9 °C of 30 s for (IL4), and 63 °C of 30 s for (VEGF), elongation stepped for 30 s at 72 °C, and the final extensions for 5 min at 72 °C. The PCR products of each gene were gel electrophoresed using 2.6% agarose with 13 μl safe stained dye DNA (Cat. no G8140), 10 μl ladder (LowRanger 100 bp DNA Ladder (100bp-2000 bp); Cat.no 1150, NORGEN BIOTIK CORP), and 10 μl PCR gene product was added, and visualized via (UV Transilluminator; BIO View) in Zheen International Hospital; Genetic Department Laboratory; Erbil, Iraq.

### 2.7 Sanger sequencing

Prior to sanger sequencing, purification of the DNA amplicon was performed by using (ExoSAP-IT^TM^ Express PCR Product Cleanup Kit, Cat. no. 75001 and 75002, USA) to remove unincorporated dNTPs, unbound primers, polymerase enzymes, salts, and other impurities that were part of the PCR reaction generating the DNA sequencing target. Then 1 μl of purified DNA was used for Sanger Reaction or Cycling by using (Z. BigDye Terminator v3.1 Cycle Sequencing Kit, Cat.no. 4337458, USA). Moreover, Sanger purification were performed using (BigDye XTerminator™ Purification Kit, Cat.no. 4376487). Finally, samples were analyzed by using (appliedbiosystems by Thermo Fisher Scientific, SeqStudio Genetic Analyzer, Germany), in Zheen International Hospital; Genetic Department Laboratory; Erbil, Iraq.

### 2.8 Statistical analysis

After performing a normality test, the statistical differences in the serological data for the CA15-3, IL-1β, IL-4, and VEGF between control and BC patients were evaluated using the U-Mann Whitney test. For analysis of the results of Sanger sequencing the Mutation Surveyor software V5.1.2 was used, the software automatically aligned sample traces to GenBank references. Data analysis was done using the program GraphPad Prism 8.0. Interquartile range and the median of the data were displayed. The statistical significance was fixed at *p < 0.05*.

## 3 Results

### 3.1 Tumor marker CA15-3 in BC and control individuals.

**Fig. 1:**
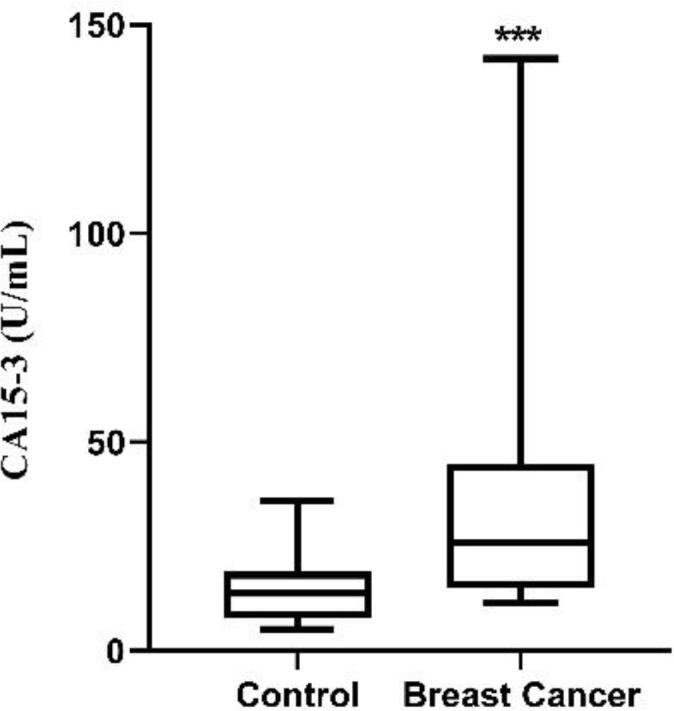
Comparison between *Tumor marker CA15-3* in BC and healthy individual. CA 15-3 level was increased significantly in patients with BC as compared with healthy control.

The concentrations of tumor marker CA15-3 in BC cases (median 26.09; range, 15.17–44.80) were increased significantly as compared with the levels of control individuals (median 13.72; range, 7.883–18.90) (*P < 0.0002*). Additionally, CA15-3 tumor marker was deemed to be an accurate biomarker for BC. (Fig. 1).

### 3.2 Serum IL-1β, IL-4, and VEGF concentrations

**Fig. 2:**
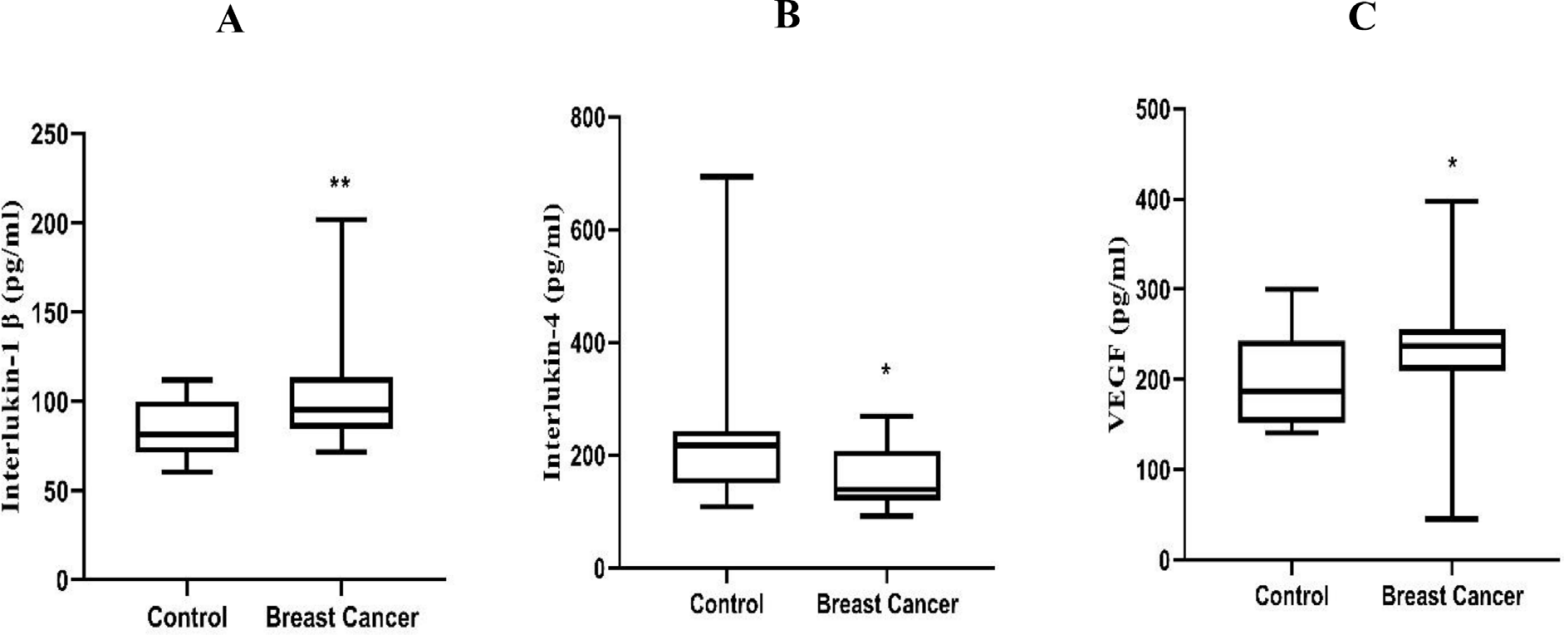
Comparison between IL-1β (pg/ml), IL-4 (pg/ml) and VEGF (pg/ml) levels in control groups and patients with BC. IL-1β IL-4 level were increased significantly in patients with BC as compared with healthy control. VEGF level was increased significantly in patients with BC as compared with healthy control.

In breast cancer, the levels of IL1β (median 95.45; range, 84.80–113.8) and VEGF (median 237.0; range, 209.5–255.6) were significantly increased compared to healthy individuals IL-1β (median 81.45; range, 71.20–100.2) and VEGF (median 187.0; range, 152.3–243.1), while BC serum concentration of IL-4 (median 139.1; range, 120.2–207.9) was significantly decreased compared with control group IL-4 (median 217.2; range, 149.9–243.2) (Fig. 2).

### 3.3 Genomic polymorphisms of IL-1β, IL-4 and VEGF.

**Fig. 3:**
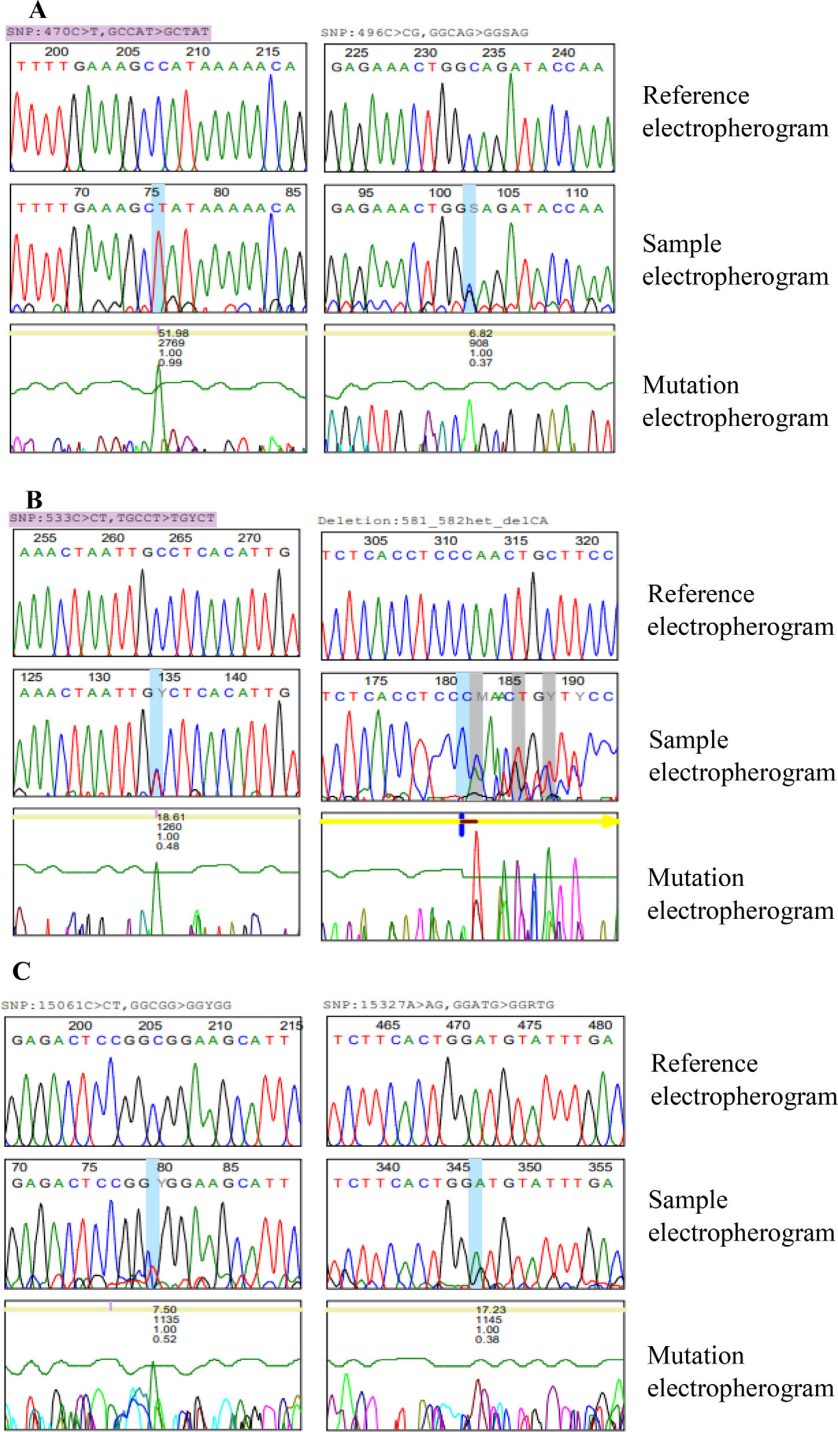
DNA sequence electrograms showing nucleotide mutations in IL-1β, IL-4 and VEGF. (A) Heterozygous substitution from C to T and heterozygous substitution from C to CG of IL-1β gene. (B) Heterozygous substitution from C to CT and heterozygous deletion 581_582CA in exon region of IL-4 gene. (C) Heterozygous substitution from C to CT and heterozygous substitution from A to AG of VEGF gene.

In Fig. 3, the rows indicate electropherograms of reference, sample and mutation sequences. Totally, 21 mutations were recorded in the 3 genes (Table 1). In IL-1β, eight mutations were identified, 10 mutations were recorded in IL-4 and three mutations were reported for VEGF. Extensively, in IL-1β, all genomic mutations belong to the nucleotide substitution type, (A > AG, A > AT, C > CT and C > CG). The heterozygous variant mutation 470C > CT,$13 on chromosome position 2:113594387 and the heterozygous variant mutation 579C > CT,$8 on chromosome position 2:113594278 have been reported previously in external public database dbSNP:1143627 and dbSNP:1143628 respectively and their variant percentage were 66.7% and 5.9%, respectively. Meanwhile, as far as we know, none of the other mutational changes of IL-1β gene have been recorded in external databases. The evaluation of IL-4 gene sequencing data showed the maximum rate of mutations among the 3 genes and two types of genetic mutations were detected: Substitutions (A > AT, A > AG, G > GA and C > CT) and deletions (T and CA). The heterozygous variant mutation 533C > CT,$35 on chromosome position 5:132009710 and the heterozygous variant mutation 610A > AG,p.L15LL,$17 on chromosome position 5:132009787 have been reported previously in external databases dbSNP:2070874 and dbSNP:2243251 respectively, and their variant percentages were 15% and 5.6%, respectively. Regarding VEGF, the obtained data revealed that all genomic mutations belong to the type of nucleotide substitution (A > AG and C > CT) and on the other hand, as far as we know, all the mutations have not been reported in external databases. However, the Sanger sequencing of control samples show no mutations in the cytokines used in this study.

**Table 1:** Heterozygous variants identified in BC patients using Mutation Surveyor software.

Gene	Chromosome location	Type	Genotype	Variant	Variant %	External database
IL-1β	2:113594413	Substitution	A > AG	444A > AG,$8	16.7	Not Found
	2:113594387	Substitution	C > CT	470C > CT,$13	66.7	dbSNP:1143627
	2:113594374	Substitution	A > AT	483A > AT,$6	4.8	Not Found
	2:113594370	Substitution	A > AT	487A > AT,$18	4.8	Not Found
	2:113594361	Substitution	C > CG	496C > CG,$6	9.5	Not Found
	2:113594342	Substitution	A > AT	515A > AT,$17	4.8	Not Found
	2:113594278	Substitution	C > CT	579C > CT,$8	5.9	dbSNP:1143628
	2:113594262	Substitution	A > AG	595A > AG,$8	8.3	Not Found
IL-4	5:132009659	Substitution	G > GA	482G > GA,$5	5.9	Not Found
	5:132009660	Substitution	A > AT	483A > AT,$10	17.6	Not Found
	5:132009662	Substitution	A > AT	485A > AT,$26	11.8	Not Found
	5:132009698	Substitution	A > AT	521A > AT,$7	5.0	Not Found
	5:132009710	Substitution	C > CT	533C > CT,$35	15.0	dbSNP:2070874
	5:132009758 _5:132009759	Deletion	CA	581_582het_delCA,$7	5.0	Not Found
	5:132009766	Deletion	T	589het_delT,$13	5.3	Not Found
	5:132009787	Substitution	A > AG	610A > AG,p.L15LL,$17	5.6	dbSNP:2243251
	5:132009814	Substitution	A > AT	637A > AT,p.G24GG,$10	5.6	Not Found
	5:132009842	Substitution	A > AT	665A > AT,p.I34IF,$21	5.9	Not Found
VEGF	6:43752506	Substitution	C > CT	15061C > CT,$8	16.7	Not Found
	6:43752750	Substitution	A > AG	15305A > AG,$5	15.4	Not Found
	6:43752772	Substitution	A > AG	15327A > AG,$17	7.7	Not Found

## 4 Discussion

CA15-3, one of the mucin glycoproteins (MUC1), is widely used as a tumor marker for the diagnosis of BC (Gaughran et al., 2020). MUC1 is overexpressed in BC cells when compared to healthy breast tissue, and the CA15-3 test could be utilized to find it in peripheral blood (Mukhopadhyay et al., 2011). An increase in the expression of MUC1 on the surface of cancer cells could speed up the metastasis of those cells because CA15-3 is involved in cell-to-cell contact and cellular adhesion (Senapati et al., 2010). Elevated CA15-3 concentrations can be identified in the early stages of BC, despite the fact that it was mostly noticed in cases of metastatic BC (Nisman et al., 2013). Many malignancies and benign disorders have high CA15-3 levels. Its main use is to evaluate how well metastatic cancer patients respond to therapy (Duffy et al., 2010). However, the effectiveness of CA15-3 in patients with BC is disputed due to a lack of organ and tumor specificity and sensitivity (Kabel, 2017). CA15-3 is often employed as a marker in the monitoring of BC during diagnosis, postsurgical surveillance of women without symptoms, and monitoring treatment in metastatic patients since it is an easily accessible, rapid, economical, easy, and quantitative test (Uygur and Gumuş, 2021).

Previous studies have shown that individuals with metastatic BC more commonly experience higher blood CA15-3 levels (Gaughran et al., 2020, Fakhari et al., 2019). In this study, CA15-3 level was raised statistically in BC in comparison to controls.

Tumor growth is aided by the angiogenesis-stimulating effects of Interleukin-1β. The growth of new, small blood vessels is crucial for the development of tumors. Microscopic *in situ* tumor lesions may take years to generate their own microcirculation. A switch known as an angiogenic promotes the rapid growth and spread of malignancies (Liotta and Kohn, 2001). It has been shown that microenvironmental IL-1 promotes angiogenesis and tumor cell invasions *in vivo* (Voronov et al., 2003).

These results were supported by the observation that IL-1-secreting tumors exhibited a higher density of tumor blood vessels and much higher VEGF release from malignant cells (Voronov and Apte, 2017). During the initial angiogenic response during the development of tumors, VEGF and IL-1 interact. The development of new blood vessels requires both IL-1 and VEGF, and they both seem to stimulate one another (Bent et al., 2018).

According to the current study, BC patients' serum levels of IL-1β were statistically higher than those of the control group. The IL-1 gene has eight different exon regions (exon number 1), and DNA Sanger sequencing data revealed numerous genetic variants, including replacement mutations in these exon regions.

Interleukin-1β (IL-1β) may boost the production of chemokines, adhesion molecules, and prostaglandins. The increased expression may trigger an inflammatory cascade that includes angiogenesis, cell chemotaxis, and adhesiveness, which could result in inflammation associated with cancer (Idris et al., 2015). There is growing evidence that inflammatory responses contribute to the growth of cancer (Singh et al., 2019). In this regard, multiple studies have found a link between ovarian cancer, gastric cancer, and IL-1 gene polymorphisms (Abbasian et al., 2018, Raza et al., 2020, Nahar et al., 2022). Additionally, numerous researches have assessed the association between BC and IL-1 gene polymorphisms (Ibrahimi et al., 2022, Al-Eitan et al., 2020, Wang et al., 2019).

The development, invasion, and metastasis of cancer are all correlated with inflammation; nevertheless, many cancer cell types are immune system-evading. Chemokines, prostaglandins, and cytokines govern and exert their influence on the inflammation of cancer (Harvey and Morgan, 2014). Among which, IL-4 has been connected to various cancer types as the main inflammatory cytokine. found to be associated with an increased risk of BC, melanoma, neck and head squamous carcinoma, prostate, small cell lung cancer, and renal cell cancer (Sanchez-Correa et al., 2013, Mytilineos et al., 2020, Zhao et al., 2020).

The serum IL-4 concentrations were decreased in BC compared with control group. These study results were inconsistent with the finding of previous studies, which showed significant higher concentration of IL-4 in sera of patients with BC than those of control groups (Zhao et al., 2020, Pang et al., 2021). Data obtained from DNA Sanger sequencing in this study indicated several mutations, including substitutions and deletions in first exon region of IL-4 gene. These results provide an interpretation for the decreased level of IL-4 in BC. Moreover, several polymorphisms related to IL-4 encoding gene were shown to be a cancer risk factor (Ibrahimi et al., 2018, Shabbir et al., 2022, Safwat et al., 2022).

One of the most important angiogenic factors in metastatic BC is VEGF (Izawa et al., 2020). According to the findings of this study, BC had significantly higher levels of VEGF than the control group. These results corroborated the findings of several other studies (Będkowska et al., 2021, Kamil et al., 2021), which suggested that VEGF may be a crucial element in promoting the growth and elevation in rising numbers of endothelial cells in the majority of patients with metastatic BC. The fact that all of the patients included in this study had just received their diagnosis and did not receive chemotherapy may help to explain why VEGF levels are rising in the serum of BC patients.

Many authors have confirmed these findings in BC since the initial research of raised serum VEGF in oncologic patients (Kondo et al., 1994), with higher levels typically identified in metastatic or advanced disease compared to early BC (Będkowska et al., 2021, Kamil et al., 2021). However, DNA Sanger sequencing data revealed three novel genetic mutations (substitution mutations) in three different exon regions (exon number 8) of the VEGF gene. These results were in contrast to the findings of the study by (Abid et al., 2021), which found no discernible difference between the levels of VEGF in sera from colorectal cancer patients and those from control groups.

The findings of this study suggest that IL-1β, IL-4, and VEGF genotype mutations may have a significant influence in the development of BC. These results may contribute to a better understanding of the molecular mechanisms of BC angiogenesis and the identification of brand-new therapeutic targets. According to prior studies, those with metastatic BC typically had increased blood CA15-3 levels (Gaughran et al., 2020, Fakhari et al., 2019). In this investigation, CA15-3 levels in BC were statistically greater than in controls.

## Declaration of Competing Interest

The authors declare that they have no known competing financial interests or personal relationships that could have appeared to influence the work reported in this paper.
